# The Genome Sequence of Lone Star Virus, a Highly Divergent Bunyavirus Found in the *Amblyomma americanum* Tick

**DOI:** 10.1371/journal.pone.0062083

**Published:** 2013-04-29

**Authors:** Andrea Swei, Brandy J. Russell, Samia N. Naccache, Beniwende Kabre, Narayanan Veeraraghavan, Mark A. Pilgard, Barbara J. B. Johnson, Charles Y. Chiu

**Affiliations:** 1 Department of Laboratory Medicine, University of California San Francisco, San Francisco, California, United States of America; 2 UCSF-Abbott Viral Diagnostics and Discovery Center, San Francisco, California, United States of America; 3 Division of Vector-borne Diseases, Centers for Disease Control and Prevention, Fort Collins, Colorado, United States of America; 4 Department of Biology, San Francisco State University, San Francisco, California, United States of America; Blood Systems Research Institute, United States of America

## Abstract

Viruses in the family *Bunyaviridae* infect a wide range of plant, insect, and animal hosts. Tick-borne bunyaviruses in the *Phlebovirus* genus, including Severe Fever with Thrombocytopenia Syndrome virus (SFTSV) in China, Heartland virus (HRTV) in the United States, and Bhanja virus in Eurasia and Africa have been associated with acute febrile illness in humans. Here we sought to characterize the growth characteristics and genome of Lone Star virus (LSV), an unclassified bunyavirus originally isolated from the lone star tick *Amblyomma americanum*. LSV was able to infect both human (HeLa) and monkey (Vero) cells. Cytopathic effects were seen within 72 h in both cell lines; vacuolization was observed in infected Vero, but not HeLa, cells. Viral culture supernatants were examined by unbiased deep sequencing and analysis using an in-house developed rapid computational pipeline for viral discovery, which definitively identified LSV as a phlebovirus. *De novo* assembly of the full genome revealed that LSV is highly divergent, sharing <61% overall amino acid identity with any other bunyavirus. Despite this sequence diversity, LSV was found by phylogenetic analysis to be part of a well-supported clade that includes members of the Bhanja group viruses, which are most closely related to SFSTV/HRTV. The genome sequencing of LSV is a critical first step in developing diagnostic tools to determine the risk of arbovirus transmission by *A. americanum*, a tick of growing importance given its expanding geographic range and competence as a disease vector. This study also underscores the power of deep sequencing analysis in rapidly identifying and sequencing the genomes of viruses of potential clinical and public health significance.

## Introduction

*Bunyaviridae* is the largest family of viruses, with over 350 species that infect a broad range of hosts including plants, arthropods, and vertebrate animals [1]. Bunyaviruses pathogenic to humans are associated with severe febrile, respiratory, and hemorrhagic diseases. These bunyaviruses include Crimean-Congo Hemorrhagic Fever (CCHF) virus [2], a tick-borne acute hemorrhagic disease in Asia, Europe, and Africa with a case fatality rate of up to 30%, and hantaviruses [3], a suite of rodent-borne diseases worldwide that are associated with pneumonia or hemorrhagic fever with renal syndrome. The *Bunyaviridae* family is comprised of five genera: *Nairovirus*, *Bunyavirus*, *Hantavirus*, *Phlebovirus*, and *Tospovirus* [4]. Their genomes consist of three single-stranded negative-sense RNA segments: large (L), encoding the L protein, an RNA-dependent RNA polymerase (RdRp); medium (M), encoding glycoproteins Gn and Gc; and small (S), encoding the nucleocapsid protein (N) as well as an ambisense nonstructural protein (NSs) in a subset of viruses.

In 2011, a new bunyavirus in the *Phlebovirus* genus, named Severe Fever with Thrombocytopenia Syndrome Virus (SFTSV), was reported as the cause of an outbreak of severe febrile illness in China [5], [6], [7]. Between 2008 and 2010, approximately 500 patients from eastern China, predominantly farmers in rural hilly areas of Hubei and Henan provinces, were diagnosed with SFTSV infection. The disease caused by SFTSV was characterized by fever, anorexia, fatigue, and depressed platelet and white cell counts [6]. Because of the similarity of the clinical symptoms of SFTS disease to those seen in human granulocytic anaplasmosis, the etiological agent was originally believed to be *Anaplasma phagocytophilum*. However, two research groups independently discovered a novel phlebovirus as the cause of SFTS [5], [6], and additional findings implicated the hard tick, *Haemaphysalis longicornis*, as the vector of SFTSV [7]. Recently, the discovery of Heartland virus (HRTV), a new, putatively tick-borne phlebovirus distinct from LSV and associated with two human cases of critical febrile illness from Missouri, was also reported [8]. Strains of Bhanja (BHAV) and Palma (PALV) virus have also been fully sequenced and found to constitute a novel clade of tick-borne phleboviruses [9], [10]. Although the pathogenic spectrum of the BHAV group viruses has not yet been fully defined, Bhanja virus has been associated with febrile illness with central nervous system involvement in both laboratory and naturally infected cases [5], [6], [7], [11], [12], [13].

In light of these studies, which have augmented interest in phleboviruses as potential agents of human disease, we sought to sequence the genome of the Lone Star virus (LSV), an unclassified bunyavirus virus originally isolated from the lone star tick, *Amblyomma americanum* [14]. The tick from which LSV was recovered had been feeding on a woodchuck in the eastern United States (Land-Between-the-Lakes area of Kentucky) in 1967. Although LSV had remained genetically uncharacterized for more than 40 years, our interest in it was piqued because exposure to the *A. americanum* tick has been associated with an illness of unknown etiology called Southern Tick-borne Rash Illness (STARI) [15], [16], [17], [18], a condition that is similar clinically to early Lyme disease. We wished to characterize LSV so that we could develop appropriate diagnostic tools to evaluate whether this virus could be found in clinical specimens from STARI patients.

Here we used next-generation sequencing coupled with *de novo* assembly to recover the highly divergent genome of LSV at >1,000-fold average coverage. We show that it is indeed a phlebovirus in the *Bunyaviridae* family and that it readily infects human and monkey cell lines in vitro.

## Results

**Figure 1:**
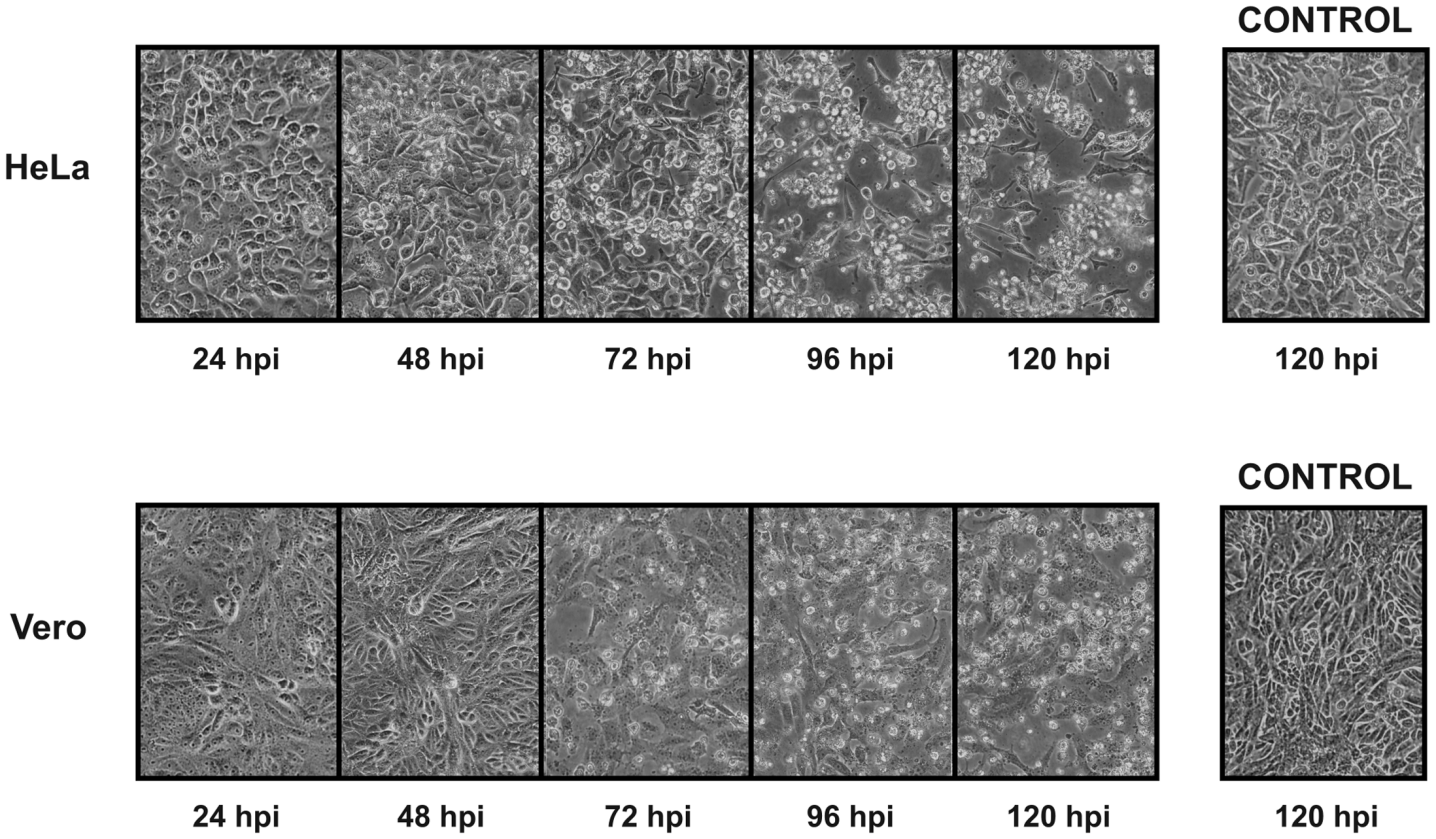
Time course of the development of cytopathic effects by Lone Star virus in human (HeLa) and monkey (Vero) cell cultures. CPE is shown at 24, 48, 72, 96, and 120 hours post-inoculation (hpi). Uninfected controls at 120 hpi are also shown.

Lone Star virus (LSV) induced cytopathic effect (CPE) in both non-human primate (Vero) and human (HeLa) cell types at 72 hours post-inoculation (hpi) (Fig. 1). Nearly complete clearing of the cell sheet was observed with infected HeLa cells, but not with Vero cells, at 96 hpi. Inoculated Vero cells developed vacuoles starting at 72 hpi with increasing abundance as CPE progressed. Vacuoles were not observed with inoculated HeLa cells. The titers of infectious LSV produced in HeLa and Vero cell culture at 72 hpi in plaque-forming units (PFU) per milliliter were 1.9×10^6^ PFU/ml and 1.2×10^6^ PFU/ml, respectively.

**Figure 2:**
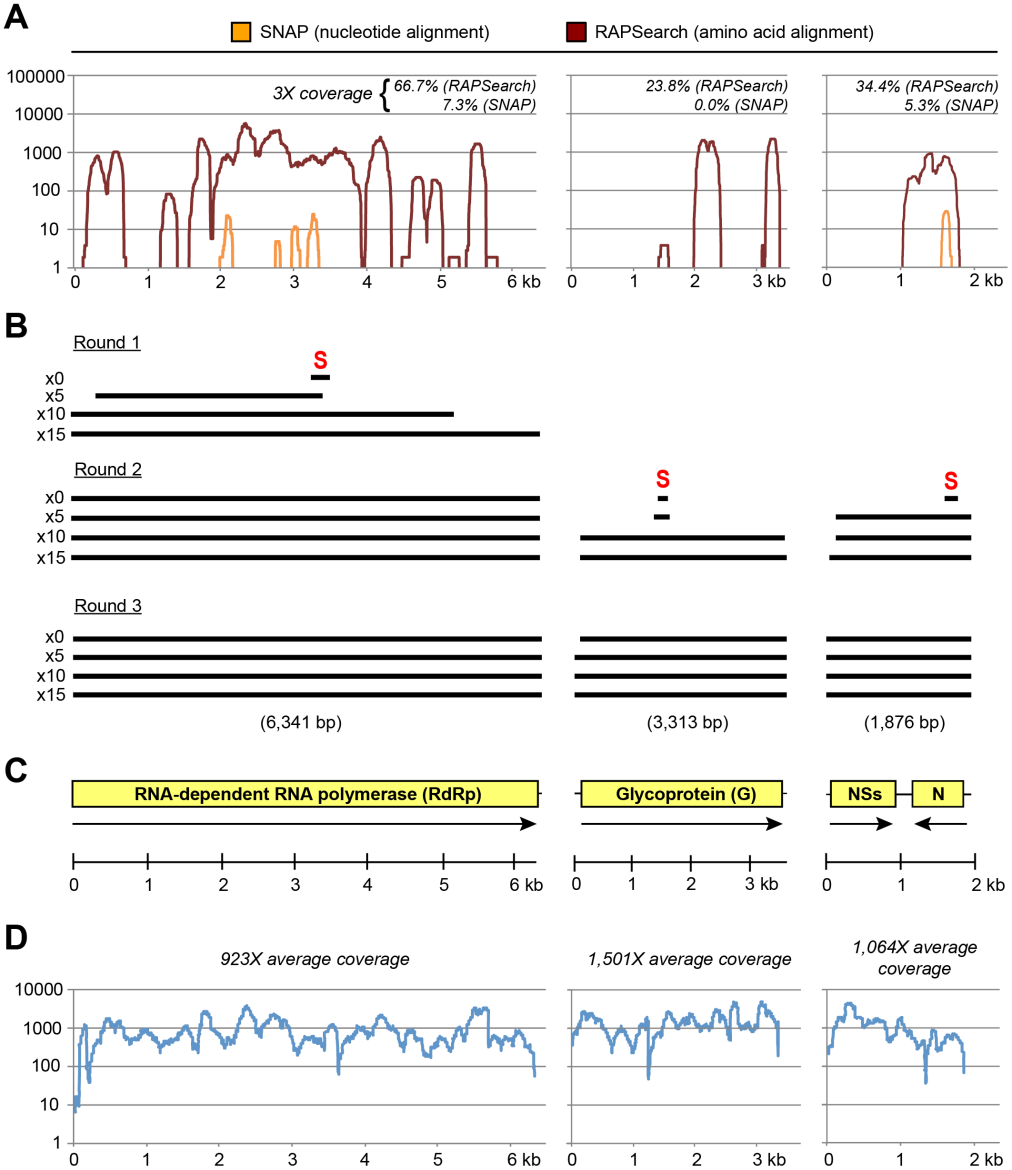
Identification and assembly of the LSV genome by unbiased deep sequencing. (**A**) Using a rapid computational pipeline, reads identified as bunyaviruses by SNAP nucleotide alignment (orange) or RAPSearch amino acid alignment (dark red) were mapped to the assembled LSV genome. The coverage (y-axis) achieved at each position along the genome (x-axis) is plotted on a logarithmic scale. (**B**) *De novo* assembly of the LSV genome using the PRICE assembler (3 rounds of 15 cycles each) and LSV seed sequences (“S”) identified from (A). (**C**) The genome structure of LSV. Boxes represent open reading frames (ORFs) corresponding to the RdRp, G, N, and NSs proteins, flanked by noncoding regions, which are indicated by lines. Coding directions are indicated by arrows. (**D**) Mapping of the actual deep sequencing reads derived from LSV to the final assembled genome. The coverage (y-axis) achieved at each position along the genome (x-axis) is plotted on a logarithmic scale. GenBank accession numbers are reported in the text. Abbreviations: kb, kilobases; bp, base pairs.

Unbiased next-generation or “deep sequencing” was then used to analyze LSV culture supernatants and assemble the viral genome. Since our standard virus purification protocol using nuclease prior to extraction resulted in a very low concentration of RNA (less than 5 ng/µL), this material was pooled with RNA from a second extraction without nuclease treatment for cDNA deep sequencing library preparation. The final count of raw deep sequencing reads was 15,134,328 total sequences (7,567,164 150-bp paired-end sequences). Using a rapid computational pipeline developed in-house for pathogen identification from deep sequencing data (Naccache, et al., manuscript in preparation), the dataset was comprehensively analyzed for viruses within 2 h using a single 64-core computational server with 512 GB RAM. First, NGS reads were sequentially preprocessed, aligned to a reference human database, and aligned to a reference bacterial database, with removal of 50.3% (n = 7,606,235), 15.5% (n = 2,345,988), and 1.9% (n = 293,410), respectively, of the raw sequences in the dataset. The remaining 4,888,695 sequences, comprising 32.3% of the original dataset, were then aligned to reference viral nucleotide and protein databases to identify reads corresponding to viruses (Table 1). Among the detected viruses, the vast majority were known contaminants in cell cultures, reagents, and the laboratory environment (Table 1). The sole exception was a phlebovirus in the *Bunyaviridae* family, reads from which comprised 92.2% of the viral deep sequencing hits. The phlebovirus reads represented all 3 segments and were highly divergent, sharing <50% identity with any other bunyavirus sequence in GenBank. As the alignable reads represented less than 50% overall coverage of the genome (Fig. 2A), the full LSV genome was subsequently recovered by 3 rounds of 15-cycle *de novo* assembly using a single “seed” corresponding to an identified read for each of the presumptive L, M, and S segments (Fig. 2B). Subsequent mapping of the preprocessed deep sequencing reads to the full LSV genome at high stringency showed that the actual coverage achieved averaged 1,112X [range 7–4,939X] (Fig. 2C). The mapping also revealed that the computational pipeline had detected only 26.1% (37,322/142,941) of the total number of LSV reads actually present in the deep sequencing data (Table 1).

**Table 1:** Deep sequencing reads matching to viral sequences.

Virus	Viral Family	# of viral reads by SNAP*	# of viral reads by RAPSearch*	# (%) of viral reads by RAPSearch+SNAP*	Presumed source
Simian endogenousretrovirus	*Retroviridae*	1,217	1,502	2,008 (5%)	Vero cell endogenous retrovirus
Avian myeloblastosis virus	*Retroviridae*	608	833	936 (2.3%)	Known viral contaminant of reverse transcriptase in ScriptSeq kit
Phlebovirus (LSV)	*Bunyaviridae*	90	37,292	37,322 (92.2%)	Culture of LSV
Bovine viral diarrhea virus	*Flaviviridae*	93	139	148 (0.37%)	Contaminant of fetal bovine serum used in virus culturing
Environmental DNA viruses	various families*	4	73	75 (0.19%)	Environmental and/or reagent contamination
TOTAL (%)		2,012	39,839	40,489 (100% of viral reads, 0.27% of total reads)	

* Viral reads were identified by comparison to viral nucleotide and amino acid databases using the SNAP [29] and RAPSearch [30] aligners, respectively. Viral hits were confirmed to be true by BLASTn alignment to LSV or the closest viral genus or species in GenBank using an E-value cutoff of 1×10^−8^. Out of 15,134,328 total reads, 40,489 reads (0.27%) were identified as viral by SNAP and/or RAPSearch. Out of these 40,489 viral hits, 37,322 (92.2%) corresponded to LSV. The actual number of LSV reads in the dataset is 142,941 (0.94% of the total reads); thus, only 26.1% (37,322 of 142,941) of the actual number of LSV reads was detected.

**Figure 3:**
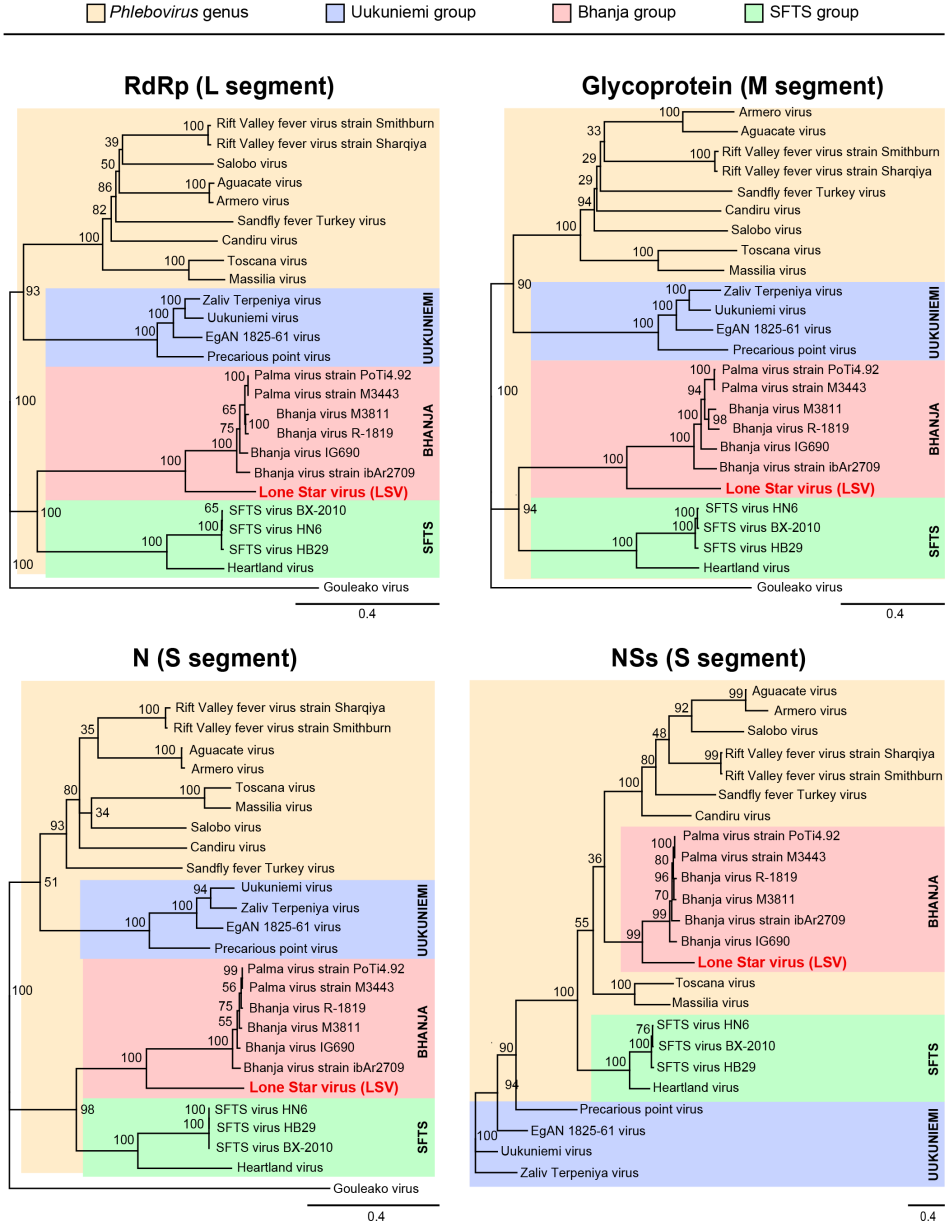
Amino acid phylogenetic analysis of the four LSV protein sequences relative to those from representative phleboviruses and Gouleako virus. For the RdRp, glycoprotein, and N protein, Gouleako virus is included as an outgroup to the phleboviruses (tan). Gouleako virus, the closest known bunyavirus relative to phleboviruses, is a member of a proposed new genus in the family *Bunyaviridae* [36]. Also shown color-coded are the Uukuniemi (blue), Bhanja (red), and SFTS (green) clades of known tick-borne phleboviruses. GenBank accession numbers are reported in the text.

As in other bunyaviruses, the genome of LSV consists of 3 negative-sense RNA segments (L, M, and S) (Fig. 2B). These segments were found to contain coding sequences for the RdRp, G, N, and NSs proteins. The sizes of the L, M, and S segments of LSV are 6,341, 3,313, and 1,876 nt, respectively, encoding 2,085 amino acid (aa) L, 1,084 aa G, 247 aa N, and 316 aa NSs proteins. By phylogenetic analysis, LSV was found to be a member of the *Phlebovirus* genus, and part of a well-supported clade containing the recently sequenced Bhanja and Palma viruses (Fig. 3, “Bhanja”) [9], [10]. This clade was distinct from the SFTS group of tick-borne phleboviruses, which includes SFTSV and HRTV (Fig. 3, “SFTS”) [5], [6], [7], and the Uukunemi group, which includes among its members the Zaliv Terpeniya, Uukuniemi, EgAN 1825–61, and Precarious point viruses (Fig. 3, “Uukuniemi”) [19], although it is more closely related phylogenetically to the SFTS than Uukunemi group.

**Figure 4:**
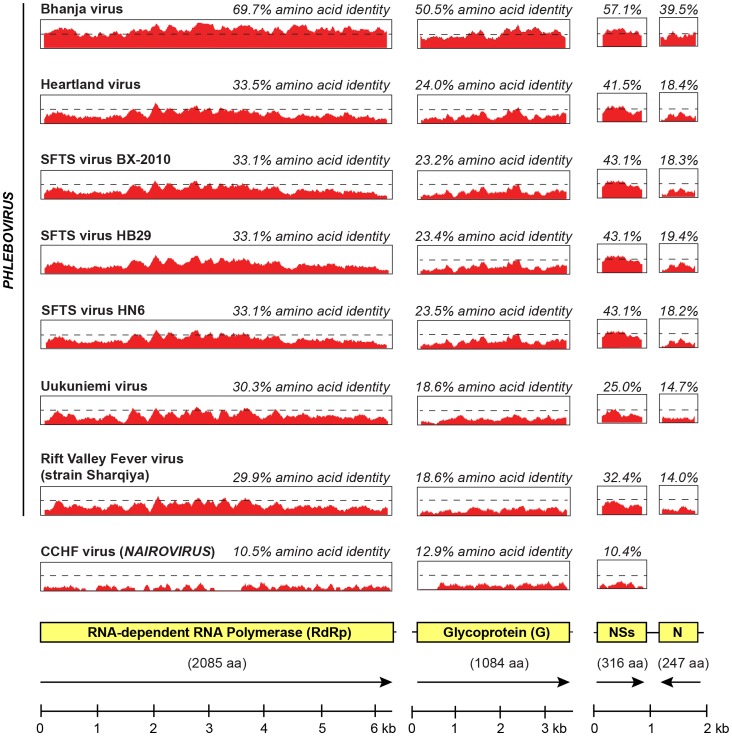
Amino acid pairwise identity of LSV relative to other representative bunyaviruses. The amino acid identities are shown for the four LSV proteins (RdRp, G, N, and NSs). A sliding window of 50 bp was used. GenBank accession numbers are reported in the text.

The termini of the L, M, and S segments of LSV retain the conserved “5-ACACAAAG” and “CUUUGUGU-3” inverted signature sequences common to phleboviruses in the *Bunyaviridae* family, with the notable exception of the 5′ end of the S segment, which contains a “5′-ACACA**G**AG” sequence. This deviation from absolute conservation of the terminal signature sequences is also seen in other tick-borne phleboviruses, including SFTS, Heartland, Bhanja, and Palma viruses [9]. Pairwise identity plots revealed that LSV is highly divergent with ≤61% overall amino acid identity to other representative bunyaviruses (Fig. 4). The closest relatives to LSV were the Bhanja and Palma viruses, with 39–70% amino acid identity across the 4 bunyavirus proteins (Fig. 4), with the next nearest neighbors, the SFTSV and Heartland viruses, sharing only 18–43% amino acid identity.

## Discussion

Phleboviruses that are pathogenic to humans can be transmitted by mosquitoes, sand flies, and ticks [6]. The *A. americanum* tick is well-suited as a zoonotic disease vector because it feeds on a wide assortment of wild animal hosts as well as humans. It is a known vector for the pathogenic agents of Rocky Mountain spotted fever (*Rickettsia rickettsia*), human monocytic ehrlichiosis (*Ehrlichia chaffeensis*), and tularemia (*Francisella tularensis*), and has also been associated with Southern Tick-borne Rash Illness (STARI), for which no etiologic agent has been identified [20]. Because the geographic range of *A. americanum* is expanding northward in the eastern United States and it has a propensity for biting humans [21], its potential role as a vector of disease is increasingly important. However, the ability of *A. americanum* to harbor viruses is not well understood. The sequencing of LSV is thus a critical first step in determining the risk of arbovirus transmission by *A. americanum*.

Our study found that LSV is a member of the Bhanja clade of tick-borne phleboviruses (Fig. 3) [10], which shares greater phylogenetic similarity to members of the SFTS than Uukuniemi clade (Figs 3 and 4). Despite high sequence divergence, serological cross-reactivity was recently observed between members of all 3 clades [10], [19]. This suggests that members of the SFTS, Bhanja, and Uukuniemi clades comprise part of a single larger serogroup of tick-borne phleboviruses. As phleboviruses in the SFTS and Bhanja clades are known to be pathogenic in humans [5], [6], [7], [11], [12], [13], LSV may also potentially be associated with tick-borne illness in humans, and further investigation is underway to explore this possibility.

In this study, both human (HeLa) and monkey (Vero) cells were found to support infection by LSV, suggesting that LSV may be capable of infecting human and other nonhuman primates *in vivo*. However, although CPE was observed in both HeLa and Vero cells starting at 72 hpi, the appearance and progression of the CPE was different between the two LSV-inoculated cell lines. In particular, vacuoles were observed only in Vero cells. Similarly, cellular vacuoles presumably containing infectious viral particles were also seen with SFSTV and Heartland virus, which also grow efficiently in Vero cells [6], [8].

Identifying pathogens and then rapidly assembling their genomes *de novo* is challenging for widely divergent sequences such as those found in novel emerging viruses. When related genomes in large sequence databases such as GenBank are lacking, routine algorithms to map/align reads to reference sequences are inadequate. To address these challenges, we have developed a computational pipeline for rapid identification and *de* novo genome assembly of viral pathogens from deep sequencing metagenomic datasets (Naccache, et al., manuscript in preparation). After preprocessing and removal of sequences corresponding to host background and/or laboratory contamination, this pipeline incorporates both nucleotide and amino acid alignments to reference databases using efficient and highly parallelizable algorithms to comprehensively identify both known and novel viruses within hours. Protein alignments are critical for detecting highly divergent viruses such as LSV, as shown by the detection of ∼414X the number of phlebovirus reads by amino acid rather than nucleotide alignments (37,292 vs. 90, Table 1). In fact, even with the use of low-stringency amino acid alignments, the computational pipeline was only able to identify 26.1% of the total number of LSV reads actually present in the deep sequencing dataset (Table 1) due to the high sequence divergence of LSV relative to other bunyaviruses (Fig. 4). Thus, for genomic assembly, downstream “seed-based” *de novo* assembly packages such as PRICE are useful for recovering full viral genomes in the absence of a closely related reference sequence (Fig. 2A) [22], [23].

The incidence of new infectious diseases continues to increase. Vector-borne diseases accounted for nearly 30% of the emerging infectious disease events in the past decade [24]. The ability to rapidly identify emerging vector-borne pathogens for surveillance or outbreak investigation will be an important part of understanding and dealing with these new diseases. Here we demonstrate a deep sequencing-based approach for the rapid identification and *de novo* sequence assembly of a highly divergent phlebovirus in the *A. americanum* tick. Given the rapidly expanding geographic range of this tick [21] and its competence as a vector of zoonotic diseases [25], the role of LSV as a potential human pathogen should be investigated more thoroughly. Further investigation is also needed to assess the possible role of *A. americanum* as a vector for phleboviruses such as LSV and Heartland virus.

## Methods

Lone Star virus strain TMA 1381 was obtained from the arbovirus reference collection of the Division of Vector-borne Diseases, Centers for Disease Control and Prevention, Fort Collins, CO. This virus was originally submitted to the CDC arbovirus catalog by Robert Kokernot (University of Texas, Houston) in 1984 (http://wwwn.cdc.gov/arbocat/index.asp). Experiments using LSV were performed in Biosafety Level-2 (BSL-2) facilities certified by the Institutional Biohazards Committees of the CDC and UCSF.

An LSV suckling mouse brain passage 5 preparation was inoculated into T25 flasks of HeLa and Vero cells cultured in DMEM medium (Invitrogen, NY) supplemented with 2% FBS (Atlas Biologicals, Fort Collins, CO). The multiplicity of infection was 1.3 pfu/cell. Cytopathic effect (CPE) was monitored at 24-hour intervals for 7 days. Viral titers were determined at 72 hours post-inoculation (hpi) using a plaque assay. Briefly, cell supernatant was diluted ten-fold and 100 µl was inoculated onto Vero cell monolayers in 6-well plates using a 0.5% agarose double overlay. The cells were visualized with neutral red added to the second overlay.

LSV-infected Vero cell cultures were extracted using two different protocols, one to purify RNA from viral particles (“virus purification protocol”) and the other to extract total RNA. First, 130 µL of viral culture supernatant was treated with Turbo DNase, Baseline Zero DNase, Benzonase, and RNase A (Roche, South San Francisco, CA), and then extracted with the QIAamp UltraSens Virus Kit (Qiagen, Valencia, CA). A second RNA extraction without pre-nuclease treatment was performed on 400 µL of supernatant using TRIzol reagent (Life Technologies, Foster City, CA). Each extraction was performed according to the manufacturer’s protocol.

An LSV cDNA library for Illumina sequencing was prepared with the ScriptSeq version 2 kit according to the manufacturer’s protocol (Epicentre, Madison, WI). The input consisted of 30 ng of RNA from the first extraction and 80 ng from the second extraction. RNA was reverse-transcribed to cDNA, adaptors were ligated to the cDNA ends, and the cDNA was then amplified with 17 cycles of PCR. AMPure XP beads (Beckman Coulter Genomics, Brea, CA) were used to remove primer and adaptor dimers as well as larger PCR fragments (>600 bp) from the amplified cDNA library. Library size distribution and concentration were determined using a High Sensitivity DNA kit on an Agilent Bioanalyzer 2100 instrument (Agilent, Santa Clara, CA) and a KAPA Library Quantification Kit (Kapa Biosystems, Woburn, MA), respectively. Approximately 10 pmol of library was used for 150-bp paired-end sequencing on an Illumina MiSeq Sequencer (Illumina, Hayward, CA).

All computational analyses of deep sequencing data were performed on a 64-core 1 U Quad AMD Opteron 6200 computational server with 512 GB RAM and using the Ubuntu 12.04 LTS operating system. Raw deep sequencing reads were first “preprocessed” by trimming of primers, filtering to exclude low-quality and low-complexity sequences, and removing all residual sequences <50 bp in length [26], [27], [28]. Preprocessed reads were then analyzed using a rapid computational pipeline incorporating the SNAP [29] and RAPSearch [30] aligners for alignment to nucleotide and protein databases, respectively (Naccache, et al., manuscript in preparation). The format of the pipeline was based on a computational subtraction approach used previously for detection of 2009 pandemic influenza A [31] and a novel hemorrhagic fever virus from Africa [32]. Briefly, nucleotide alignments to human (hg19) and bacterial databases in GenBank were performed at high-stringency cutoffs (edit distance d12 for SNAP) to exclude sequences corresponding to host and protein alignments to the viral GenBank database at low-stringency cutoffs (edit distance d28 for SNAP and 10^−1^ for RAPSearch) to identify viral sequences. For these initial alignments, the advantage of SNAP and RAPSearch was a 10–1,000X increase in computational speed while maintaining comparable accuracy relative to existing algorithms such as BLASTn/BLASTx [29], [30], [33]. Candidate viral sequences were subsequently confirmed as true by direct BLASTn alignment to the identified viral genus or species using an E-value cutoff of 1×10^−8^. The approximate computational times for the preprocessing, SNAP nucleotide alignment to human/bacterial/viral databases, and RAPSearch amino acid alignment to the viral protein database were 5 minutes, 10 minutes, and 135 minutes, respectively (2 h total time).

After running the deep sequencing reads through the computational pipeline, analysis of a subset of reads identified LSV as a highly divergent bunyavirus (Table 1). Coverage of the identified bunyavirus reads was not sufficient for genome assembly (4,188 of 6,341, 820 of 3,313, and 652 of 1,867, or 66.0%, 24.8%, and 34.9% of the L, M, and S segments at 3X coverage depth) (Fig. 2). Thus, a sequence “seed” was chosen from each of the presumptive L, M, and S segments and iterative *de novo* contig assembly using the entire preprocessed dataset was performed using the PRICE assembler (approximately 6 h total time) [22], [23]. Computational finishing of large contigs was then done manually using Geneious software v6.0 [34]. The sequence of the 3′ end of the L segment of LSV, assembled with only 7X coverage (Fig. 2D), was confirmed using RACE (rapid amplification of cDNA ends)-PCR [35].

Phylogenetic analysis was performed on the 4 bunyavirus proteins: RdRp (L segment), glycoprotein (M segment), N protein (S segment), and NSs protein (S segment). Multiple sequence alignments of each of the LSV proteins relative to the corresponding protein from nearly all available phlebovirus sequences in GenBank and from Gouleako virus [36] were first generated using MAFFT (v6.0) with the E-INS-I algorithm and at default settings [37]. Gouleako virus, a member of a novel bunyavirus genus and the closest known relative to phleboviruses [36], was chosen as the outgroup for the RdRp, glycoprotein, and N protein trees. Phylogenetic trees and bootstrap confidence levels after 10,000 bootstrapping replicates were determined in Geneious using a Jukes-Cantor model and the neighbor-joining method at a support threshold of 25% (Fig. 3). Tree topologies were then confirmed using an alternate maximum likelihood Bayesian approach with MrBayes V3.2 software (20,000 sample trees, 25% of trees discarded as burn-in) [38]. The overall tree topologies using neighbor joining or maximum likelihood approaches were the same (data not shown).

Deduced amino acid sequences of LSV were compared by pairwise sliding window alignments in Geneious [34]. Specifically, LSV was aligned to Bhanja virus strain IG690, Heartland virus, the three SFTS phlebovirus isolates from China (SFTS BX-2010, SFTS virus HB29, and SFTS virus HN6), Uukuniemi virus, Rift Valley Fever virus strain Sharqiya, and CCHF virus, using MAFFT (v6.0) with the FFT-NS-I x1000 algorithm at default settings [37], and pairwise identities were plotted across each viral segment using a sliding window of 50 amino acids. Overall amino acid pairwise similarity was determined by concatenating the 4 bunyavirus protein sequences and running pairwise alignments in Geneious.

Genbank accession numbers used for Figs. 3 and 4 are as follows: **RdRp (L segment):** Aguacate virus (NC_015451), Armero virus (HQ661805), Bhanja virus strain ibAr2709 (JX961616), Bhanja virus strain IG690 (JX961619), Bhanja virus strain M3811 (JQ956376), Bhanja virus strain R-1819 (JX961622), Candiru virus (NC_015374), CCHF virus (NC_005301), EgAN 1825-61 virus (HM566159), Gouleako virus (HQ541738), Heartland virus (JX005847), Massilia virus (EU725771), Palma virus strain PoTi4.92 (JX961628), Palma virus strain M3443 (JQ956379), Precarious point virus (HM566181), Rift Valley fever strain Sharqiya (NC_014397), Rift Valley fever virus strain Smithburn (DQ375430), Salobo virus (HM627185), Tosacana virus (NC_006319), Sandfly fever Turkey virus (NC_015412), SFTS virus BX-2010 (JF682773), SFTS virus HB29 (NC_018136), SFTS virus HN6 (HQ141595), Uukuniemi virus (UUKLRNAP), Zaliv Terpeniya virus (HMX66191); **glycoprotein (M segment):** Aguacate virus (NC_015450), Armero virus (HQ661806), Bhanja virus strain ibAr2709 (JX961616), Bhanja virus strain IG690 (JX961620), Bhanja virus strain M3811 (JQ956377), Bhanja virus strain R-1819 (JX961620), Candiru virus (NC_015373), EgAN 1825-61 virus (HM566158), CCHF virus (NC_005300), Gouleako virus (HQ541737), Heartland virus (JX005845), Massilia virus (EU725772), Palma virus strain PoTi4.92 (JX961629), Palma virus strain M3443 (JQ956380), Precarious point virus (HM566179), Rift Valley fever strain Sharqiya (NC_014396), Rift Valley fever virus strain Smithburn (DQ80193), Salobo virus (HM627183), Tosacana virus (NC_006320), Sandfly fever Turkey virus (NC_015411), SFTS virus BX-2010 (JF682774), SFTS virus HB29 (NC_018138), SFTS virus HN6 (HQ141596), Uukuniemi virus (UUKGPM), Zaliv Terpeniya virus (HMX66193); **N (S segment):** Aguacate virus (NC_015452), Armero virus (HQ661807), Bhanja virus strain ibAr2709 (JX961618), Bhanja virus strain IG690 (JX961621), Bhanja virus strain M3811 (JQ956378), Bhanja virus strain R-1819 (JX961624), Candiru virus (NC_015375), CCHF virus (NC_005302), EgAN 1825-61 virus (HM566160), Gouleako virus (HQ541736), Heartland virus (JX005843), Massilia virus (EU725773), Palma virus strain PoTi4.92 (JX961630), Palma virus strain M3443 (JQ956381), Precarious point virus (HM566180), Rift Valley fever strain Sharqiya (NC_014396), Rift Valley fever virus strain Smithburn (DQ80157), Salobo virus (HM627184), Tosacana virus (NC_006318), Sandfly fever Turkey virus (NC_015413), SFTS virus BX-2010 (JF682775), SFTS virus HB29 (NC_018137), SFTS virus HN6 (HQ141597), Uukuniemi virus (UUKNNSA), Zaliv Terpeniya virus (HMX66192); **NSs (S segment):** Aguacate virus (NC_015452), Armero virus (HQ661807), Bhanja virus strain ibAr2709 (JX961618), Bhanja virus strain IG690 (JX961621), Bhanja virus strain M3811 (JQ956378), Bhanja virus strain R-1819 (JX961624), Candiru virus (NC_015375), EgAN 1825-61 virus (HM566160), Heartland virus (JX005843), Massilia virus (EU725773), Palma virus strain PoTi4.92 (JX961630), Palma virus strain M3443 (JQ956381), Precarious point virus (HM566180), Rift Valley fever strain Sharqiya (NC_014396), Rift Valley fever virus strain Smithburn (DQ80157), Salobo virus (HM627184), Tosacana virus (NC_006318), Sandfly fever Turkey virus (NC_015413), SFTS virus BX-2010 (JF682775), SFTS virus HB29 (NC_018137), SFTS virus HN6 (HQ141597), Uukuniemi virus (UUKNNSA), Zaliv Terpeniya virus (HMX66192).

The complete genome sequence of LSV has been submitted to GenBank (GenBank accession numbers KC589005-KC589007). Deep sequencing reads have been submitted to the NCBI Sequence Read Archive (GenBank accession number SRP018532).
